# A babel of web-searches: Googling unemployment during the pandemic

**DOI:** 10.1016/j.labeco.2021.102097

**Published:** 2022-01

**Authors:** Giulio Caperna, Marco Colagrossi, Andrea Geraci, Gianluca Mazzarella

**Affiliations:** European Commission, Joint Research Centre

**Keywords:** Unemployment, Nowcast, Random forest, Covid-19, Google trends, Difference-in-Differences

## Abstract

Researchers are increasingly exploiting web-searches to study phenomena for which timely and high-frequency data are not readily available. We propose a data-driven procedure which, exploiting machine learning techniques, solves the issue of identifying the list of queries linked to the phenomenon of interest, even in a cross-country setting. Queries are then aggregated in an indicator which can be used for causal inference. We apply this procedure to construct a search-based unemployment index and study the effect of lock-downs during the first wave of the covid-19 pandemic. In a Difference-in-Differences analysis, we show that the indicator rose significantly and persistently in the aftermath of lock-downs. This is not the case when using unprocessed (raw) web search data, which might return a partial figure of the labour market dynamics following lock-downs.

## Highlights

• We contribute to the literature using web searches to study economic phenomena.
• We propose a data-driven method to exploit web searches in a cross-country setting.
• Starting from raw web searches, we create an unemployment-related indicator.
• Such indicator rose significantly and persistently in the aftermath of lock-downs.
• Web searches are well suited to study sudden labour market shocks.

## 1 Introduction

Starting with the seminal contribution of Choi and Varian (2012), Google search data proved useful to proxy a variety of economic indicators. Götz and Knetsch (2019) use Google data to forecast German GDP; Vosen and Schmidt (2011) and Vosen and Schmidt (2012) focus on forecasting consumption in, respectively, US and Germany. Focusing on financial markets, Da et al. (2015) use Google search data to build an investment sentiment index to predict different US aggregate market indices, while Hamid and Heiden (2015) create a proxy for investors attention to predict stock market volatility. (Koop and Onorante, 2019) show how Google search data can be used to improve nowcast of different macroeconomic variables in the context of dynamic model selection. Finally, focusing on unemployment, D’Amuri and Marcucci (2017) assess the performance of Google search data related to job-search in forecasting US monthly unemployment rate.^1^

Google searches are particularly attractive in those contexts in which data about the phenomenon of interest are either not available or available at a low time-frequency. Further, compared to surveys, Google searches are less sensitive to the small-sample bias (Baker and Fradkin, 2017). This two features made web searches an ideal source of data for researcher during the covid-19 pandemic. For example, scholars have used Google searches to predict the number of unemployment insurance (UI) claims in the US (Aaronson, Brave, Butters, Sacks, Seo, 2020, Borup, Rapach, Schütte, et al., Goldsmith-Pinkham, Sojourner, 2020, Larson, Sinclair, 2021). Other applications (e.g., Brodeur, Clark, Flèche, Powdthavee, et al., 2020, Fetzer, Hensel, Hermle, Roth, 2020) used Google Trends data to investigate the impact of lock-downs on well-being or economic anxiety. Brunori and Resce (2020) showed instead how web queries related to symptoms can be used to monitor the diffusion of the virus.

The use of online searches crucially hinges on their association with the underlying phenomenon of interest. This, in turn, translates into the researchers’ ability to identify the most relevant set of queries in a given language and institutional context. This task is particularly challenging in a cross-country setting, where finding an ad-hoc list of keywords is either costly (in terms of time) or not feasible (due to language barriers).

In this paper, we propose a data-driven procedure to retrieve, validate and identify a set of Google Trends queries which are linked to an underlying economic phenomenon of interest. This set of queries can then be combined to construct an indicator which, in turn, can be used for causal inference. We apply this procedure to estimate the impact of containment measures on unemployment-related web searches during the first wave of the covid-19 pandemic in the EU27.

**Fig. 1:**
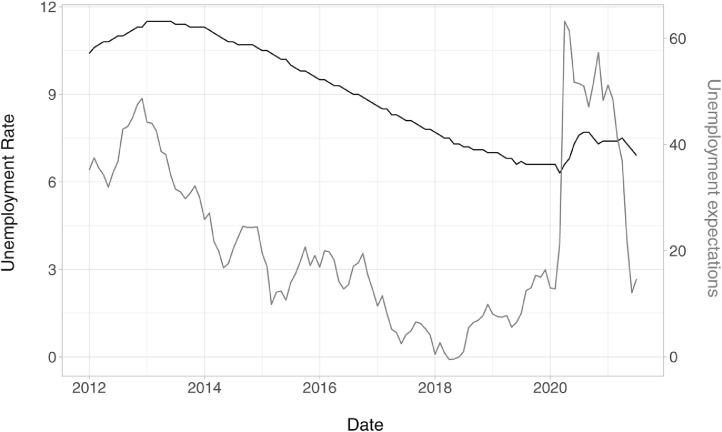
Unemployment: rate and expectations. *Note:* the left y-axis represents the EU27 seasonally adjusted monthly unemployment rate (*ei_lmhr_m*, Eurostat); the right y-axis represents the EU27 seasonally adjusted monthly indicator of unemployment expectations (*ei_bsco_m*, Eurostat). The unemployment expectations indicator is from the *Business and consumer surveys* by DG ECFIN, European Commission. The indicator is created on a monthly basis upon the replies given to the question: *How do you expect the number of people unemployed in this country will change over the next 12 months?*. The resulting indicator is a weighted balance of positive and negative answers. See https://ec.europa.eu/info/sites/default/files/bcs_user_guide.pdf.

Fig. 1 shows the EU official statistics for the unemployment rate vis-á-vis trends in expectations about future (i.e., one year ahead) unemployment levels in the EU27 before and during the first wave of the covid-19 pandemic. EU official statistics for the unemployment rate (and its expectations) are usually released with a two to three months delay and their frequency is, at best, monthly. This can be particularly challenging when facing a sudden labour market shock such as the one induced by the pandemic. Moreover, these figures – especially those on the unemployment rate – might provide a partial picture of labour markets. Several countries have implemented temporary lay-off schemes or laws suspending the right of permanently lay-off workers. Finally, official statistics for the unemployment rate are backward rather than forward-looking, a characteristic particularly relevant in a crisis such as that brought about by the covid-19 pandemic.

**Fig. 2:**
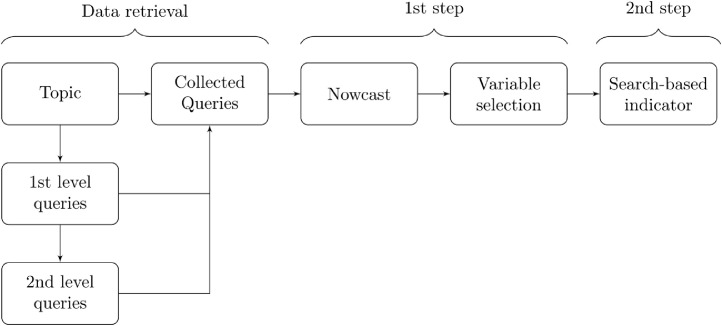
From Google topics to search-based indicators: a two-step procedure. *Note:* two-step procedure flowchart. Details about data retrieval are outlined in Section 2. The nowcast and variable selection methods (first step) as well as the construction of the indicator (second step) are discussed in Section 3.

We present a simple conceptual framework linking unemployment-related web searches to current unemployment levels and expectations. Based on this framework, we argue that unemployed-related web search behaviour could be a good real-time predictor of current and future labour market conditions. We face the challenge of identifying the correct set of keywords for each EU country. Google Trends topics, which are aggregations of different queries belonging to the same semantic concept, being language-independent, are the ideal candidates for this purpose. However, the algorithm generating topics is Google’s proprietary information, thus a black-box to researchers. In this paper, we propose to use the topic *unemployment* to collect, for each country, the entire set of language-specific associated queries in a given time-span (1st level queries) and all the top queries linked to the latter (2nd level queries). Then, as aforementioned, we develop an ad-hoc two-step procedure to construct a search-based unemployment indicator – see Fig. 2.

In the first step, we nowcast, separately for each country, the monthly unemployment rate using the Search Volume Index (SVI hereafter, see Section 2) of the collected queries. We show that nowcasting using the topic alone does not provide a statistically significant improvement over what a simple ARMA model would predict for the vast majority of countries considered (Section 3). Instead, once we add all the queries linked to the topic and introduce variable selection algorithms, the predictive accuracy increases significantly in virtually all countries.

In the second step, we select the country-specific queries that best predict unemployment rates and aggregate them to create a daily indicator of unemployment-related searches. The indicator is built, separately for each country, as the linear projection of the daily SVI of the topic on the daily SVIs of the set of best predictors.

Finally, we use the search-based indicator as the dependent variable in a Difference-in-Differences (DiD) analysis. Following the lock-down measures imposed by some EU governments to limit the spread of the SARS-CoV-2 virus, unemployment-related searches rose significantly compared to their pre-pandemic average. The higher level of searches persists throughout the lock-down period. This is not the case if using the topic “unemployment” as dependent variable. Comparing our results with those obtained using official statistics, we argue that the constructed daily indicator of unemployment-related searches better mirrors the underlying labour market dynamics.

Importantly, the data-driven procedure outlined in this paper is not only relevant in the context of the covid-19 pandemic and unemployment. It could be easily adapted to study a variety of events, policies and economic indicators.

The remainder of this paper is structured as follows: Section 2 briefly introduces Google search data. Section 3 describes our two-step procedure. Section 4 shows the results of the DiD using the indicator of unemployment-related searches. Section 5 concludes.

## 2 Google searches

Google Trends (https://trends.google.com/trends/) provides access to the search requests made to the Google search engine by its users. In particular, Google Trends contains a random sample representative of all queries that Google handles daily.^2^ Search results are normalized to the time and location of a query. By time range (either daily, weekly or monthly) and geography (either country or ISO 3166-2), each data point is divided by the total searches to obtain relative popularity. The resulting numbers are then scaled on a range of 0 to 100 based on a query’s proportion to all searches on all queries. Following the literature, we refer to this quantity as the SVI.

Google Trends returns the SVI of either queries or topics. The former are the actual search queries input by users on the Google search engine. Topics are instead aggregations of different queries that could be assigned to a particular semantic domain (in our case, unemployment). Aggregation is done by Google using semantic integration algorithms in the context of the Google knowledge graph.^3^

Topics provide few advantages over simple queries. First, since topics are language-independent, it is possible to use them to perform cross-country analyses, whereas the same does not apply to keywords. Evidence shows that search terms related to the same topic vary across countries due to cultural and institutional differences (Bousquet et al., 2017). Further, searches linked to topics might vary across time. This is particularly true for searches related to unemployment, which might also depend on the name and the seasonality of each country-specific policy. All queries broadly related to a topic are then linked to it independently from the spelling and the wording of the associated queries. In addition, Google Trends also returns the top-25 (when available) queries and topics related to any given topic or query. Top queries and topics are queries (or topics) that are most frequently searched by users within the same session for any given time and geography.

Recently, Google Trends topics have been used by Brodeur et al. (2020) to estimate the impact of lock-downs on well-being. Fetzer et al. (2020) instead use topics to measure the degree of economic anxiety during the pandemic. We take a different approach and exploit the topic both for its SVI (as done in the recent literature) and to retrieve associated queries in their native languages.

We collect the monthly SVI for the topic “unemployment” for each country for the period January 2013–December 2019. We then collect, for the same period, the monthly SVI of the “level-1” queries (i.e., the top-25 related search terms associated with the topic) and the monthly SVI for “level-2” queries (i.e., the 10-top related search terms associated with level-1 queries). For the DiD (Section 4) we instead retrieve the daily SVI of both the topic and the subset of queries we identify as the best predictors of unemployment rates in each country (Section 3) from the 13th of January to the 30th of May 2020.^4^ Each data collection procedure is then repeated five times across five days to draw values from five different sample of searches, which are then averaged. This allows obtaining more precise data (Stephens-Davidowitz and Varian, 2014), particularly for smaller countries and less common search terms.^5^

Of course, Google searches have some limitations. While 90% of EU27 household have internet access, younger individuals are more likely to use the internet than the elderly. Further, access to the internet is not random with respect to socio-economic status.^6^ While the former is a lesser concern in our case, as we do not expect the elderly to look for unemployment related-queries given that they are likely to be retired, the latter might impact our results. In particular, if low socio-economic status individuals are excluded from the queries sample, both the nowcast and the causal analysis could be downward biased.

## 3 From Google queries to an indicator of unemployment

To understand the relationship between Google searches and unemployment, we start with a simple and stylized conceptual framework. We assume an economy in which, at any given time, the amount of unemployed individuals is given by:

(1) $$U_{t}=U_{t-1}-O_{t-1,t}+I_{t-1,t}=U_{t-1}-O_{t-1,t}+{\tilde{\delta }}_{t-1,t}E_{t-1},$$

where $O_{t-1,t}$ and $I_{t-1,t}$ represent, respectively, the outflows and inflows from and in unemployment. ${\tilde{\delta }}_{t-1,t}$ is the true probability of employed individuals E at time t−1 to become unemployed at time t. We then assume the existence of a latent variable $\omega_{t}^{*}$ representing the volume of online activities related to unemployment at time t:

(2) $$\omega_{t}^{*}=\tau U_{t}+\phi E_{t}+\eta_{t}=\tau U_{t}+\tau \left({\tilde{\delta }}_{t,t+1}+\epsilon_{t}\right)E_{t}+\eta_{t},$$

where τ is the volume of online activities performed by the average unemployed individual to retrieve unemployment-related information. We assume that also employed individuals engage in such activities. Their volume ϕ is the same of unemployed individuals, τ, scaled by their (subjective) expectation of becoming unemployed in the next period ($\delta_{t})$. The relationship between the expectation and the true probability is given by the error model $\delta_{t}={\tilde{\delta }}_{t,t+1}+\epsilon_{t}$. Finally, $\eta_{t}$ is a residual term capturing online behaviour of those neither in employment nor unemployment.

In this simple representation, however, the volume of online activities related to unemployment carries information about the level of unemployment at time t – via $\tau U_{t}$ – and t+1 – via $\tau \left({\tilde{\delta }}_{t,t+1}+\epsilon_{t}\right)E_{t}$. This implies that the latent variable $\omega_{t}^{*}$ could be used in real-time to nowcast and forecast labour market dynamics.

The main challenge is to identify the set of Google search queries that best describe the latent variable $\omega_{t}^{*}$. To do so, in the first step of our procedure, we follow previous literature (e.g., D’Amuri, Marcucci, 2017, Fondeur, Karamé, 2013, Niesert, Oorschot, Veldhuisen, Brons, Lange, 2020, Smith, 2016) and perform a nowcast exercise in which the dependent variable is the monthly unemployment rate time series for each EU27 country using data from January 2013 to December 2019.^7^ Although this exercise is of interest in itself, we use it to identify the queries that best predict unemployment dynamics in each EU27 country.

Previous research nowcasting labour market dynamics using web searches adopts different strategies to identify the queries of interest. D’Amuri and Marcucci (2017) exploit the use of logical operators in the Google Trend platform, and identify the SVI associated to all queries containing the word “jobs”. Fondeur and Karamé (2013) use the single term “emploi”. Smith (2016) uses a different approach based on the root term “redundancy”. The root query is used to obtain the associated queries, and the relative volume data are aggregated using weights to produce a composite “Google Redundancy Index”. Borup et al. (2020a) show that using a set of queries rather than a single one improves out-of-sample prediction of unemployment growth in the US.

An ad-hoc choice of keywords is not feasible in our context since it would require the identification of the words which semantically define the unemployment concept in each European country. We exploit the Google topic “unemployment” to retrieve, separately for each country, the top-25 level-1 queries and the top-10 level-2 queries in the original language in the period January 2013–December 2019. This data-driven approach is similar to the use of a list of root keywords in Da et al. (2015) and Smith (2016) to retrieve the associated queries. Our root, however is not a single keyword or a list of keywords, but the language-independent topic.

After retrieving the full list of associated queries, we extract their SVI in the interval January 2013–December 2019 as well as the SVI of the topic itself. We retrieve monthly Google search data to match the unemployment rate (*ei_lmhr_m*) times series for each EU27 Member State from Eurostat.

The number of associated keywords retrieved in each country, after removing duplicates, varies from 15 (Cyprus) to 382 (Germany), with a mean of 173.7 and a median of 174.^8^ For each country we estimate different nowcast models which can be summarized as:

(3) $$\Delta u_{t}=f\left(\Delta K_{t},\Delta K_{t-1},\Delta u_{t-1},\Delta u_{t-2},\Delta u_{t-3}\right)+\varepsilon_{t},$$

where $\Delta u_{t}$ is difference of the unemployment rate between month t and t−1, $\Delta K_{t}$ is a $P_{c}$-vector comprising the differences of the monthly SVI for the P keywords retrieved for country c, including the SVI of the topic (k1 hereafter). $\Delta K_{t-1}$ is simply the lag of $\Delta K_{t}$. Finally each model includes three lags of the dependent variable. The models considered differ by the target function f(·), which maps the available information at time t to the dependent variable, as well as the number of keywords included in $K_{t}$ and $K_{t-1}$.

We evaluate the performance of each model using Pseudo-Out-of-Sample prediction (POOS hereafter) based on a rolling window framework with increasing length starting from the first 48 months. The procedure can be summarized as follows: a) the models are trained using the first 48 observations; b) the trained models are used to obtain the prediction for the 49th month; c) the models are then re-trained using the first 49 observations and predictions for the 50th are computed. The entire procedure is iterated separately for each country until month T−1.

We consider eight different models. LM1 is a classical linear ARMA(p,q) model which makes no use of Google search data and where p and q are selected based on the AIC criterion. LM2 differs from LM1 because of the inclusion of k1 in $\Delta K_{t}$ and $\Delta K_{t-1}$.

In most of the countries considered, the dimension of the time series is quite small with respect to the number of predictors, a high-dimensional context with T<<P. As an example, we retrieved 382 keywords plus the topic in Germany (which became 764 covariates when considering also their lags) compared with 80 data-points in the unemployment monthly time series.^9^ To deal with high-dimensionality, we consider two alternative models, one linear – LASSO – and one non-linear – Random Forest.^10^ LASSO_k1 and RF_k1 use the same set of information used in LM2. We use this two models to understand if potential improvements in predictive accuracy in LASSO and RF models can be driven by functional forms rather than a larger information set. LASSO_ALL and RF_ALL exploit the full set containing the SVI of all country-specific associated queries. The last two models that we consider – LASSO_LASSO and RF_BORUTA – introduce an intermediate variable selection step before starting the rolling window training. This step is performed on the entire set of observations available in each country. In the first model, the selection is done using the implicit shrinkage of LASSO, while in the second model selection is performed using the Boruta algorithm (Kursa, Rudnicki, et al., 2010, Stoppiglia, Dreyfus, Dubois, Oussar, 2003).^11^ After the selection step, we train a LASSO and a RF model using only the selected predictors obtained, respectively, using the LASSO and Boruta selection step.

The introduction of a selection step in machine learning algorithms has two objectives. On the one hand it is aimed at reducing noise due to highly correlated or redundant predictors. On the other hand, the identification of relevant predictors is useful in itself for interpretation purposes. In our context, the selection step is a way to solve the problem of identifying the most relevant set of country-specific keywords.^12^ This is similar in spirit to the procedure adopted by Da et al. (2015) to construct their index of investor sentiment starting from the volume of queries related to households economic concerns. Da et al. (2015) use as root a selected set of keywords taken from annotated dictionaries which express negative and positive economic sentiments. Götz and Knetsch (2019) also employ different variable selection methods to identify the set of keywords to be embedded in their GDP forecast models, including principal component analysis, partial least squares, LASSO and boosting.

Having obtained the time series of POOS predictions for each country, we compare the performance of the nowcast models using two different methods. In the first one, we assess the accuracy of each model against a benchmark model (LM1) using the standard one-sided Diebold-Mariano (DM) test (Diebold and Mariano, 1995). In the second case, we jointly test the accuracy of all models using the Model Confidence Set (MCS, Hansen et al., 2011) with a level of significance α=0.5.^13^ Both tests are based on absolute deviations.^14^

**Fig. 3:**
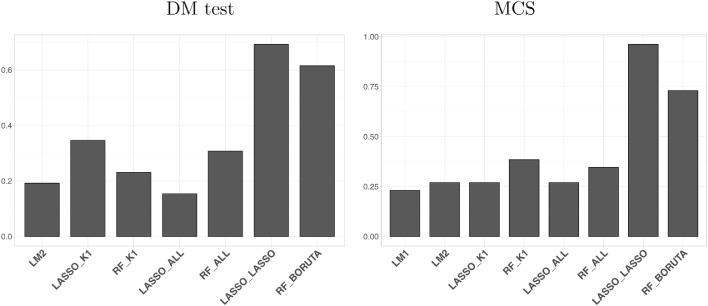
Comparing predictive accuracy of the models considered using DM test and MCS. *Note:* In the left plot, each bar represents the fraction of countries in which model i has a significantly higher predictive accuracy than the benchmark ARMA model considered, based on a one-sided Diebold-Mariano test. In the right plot, each bar represents the faction of countries in which the model is retained in the final set according to MCS (α=0.5).

Figs. 3 summarizes the main findings. In the left plot, each bar represents the fraction of *DM-victories* of each model against the benchmark LM1 across the countries considered. A model *wins* over the benchmark if its predictive accuracy is significantly higher (α = 0.1). In the right plot, each bar represents the fraction of countries for which the given model is retained in the final equally predictive set. Tables A.3 and A.4 in Appendix contain the full set of results for, respectively, DM test and MCS.

The results of the comparison show that the predictive accuracy is quite similar except for LASSO_LASSO and RF_BORUTA. In both cases, the introduction of an intermediate variable selection step leads to a sizable accuracy gain.

Strikingly, neither the inclusion of the topic alone nor of the full set of associated keywords does lead to an increase in predictive performance with respect to simpler models. This is likely due to the inclusion of a big set of noisy and redundant predictors unrelated to the true phenomenon of interest.

The drastic increase in predictive performance with the inclusion of variable selection step indicates, instead, that it is possible to identify a subset of relevant keywords which helps to reduce noise and improve nowcast accuracy. Combining the use of topics and the variable selection step in our nowcast framework presents two main advantages. On the one hand, the use of a common Google topic allows to retrieve a broad set of keywords in a context of heterogeneous countries with different languages and institutions. On the other hand, the variable selection step allows us to identify the subset of keywords which are relevant for the underlying economic variable of interest.

**Fig. A.3:**
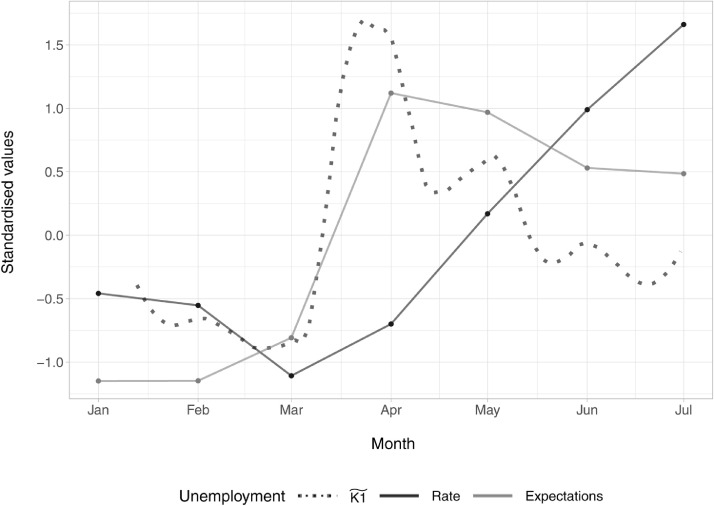
$\tilde{k1}$ indicator and official statistics. *Note:* The graph shows the standardised values of the constructed daily indicator (smoothed through local polynomial regression) using the Boruta algorithm to select the keywords (dotted line); the monthly unemployment rate (dark grey line); and monthly unemployment expectations (light grey line) during the first wave of the pandemic (January–July 2020). All points correspond to population-weighted EU averages.

Drawing from these results, in the last step of the proposed procedure, we construct two search-based unemployment indicators using the daily SVIs of the identified best predictors. $\hat{k1}$ is obtained using the predictors identified by the LASSO_LASSO procedure; and $\tilde{k1}$ by the RF_BORUTA algorithm. When constructing the indicators, we move from monthly to daily data to exploit the full potential of web search data which, contrary to official statistics, are available with daily frequency. This allows us to fully exploit the variation in lock-down dates to asses the impact of labour market shocks.^15^ Given the substantial number of web queries selected by the RF_BORUTA and (in particular) LASSO_LASSO procedures, the two indicators are created by performing, by country, a LASSO regression of the daily SVI of the country-specific subset of best predictors projected on the daily SVI of the topic. Intuitively, the indicators contain, in our application, the component of the topic explained by the keywords that best predict the unemployment rates. Fig. A.3, in the Appendix, compares the evolution of the $\tilde{k1}$ indicator with official statistics for unemployment rates and expectations before and during the first wave of pandemic (January–July 2020). It shows that the indicator quickly reacts to the introduction of lock-down measures and then remains higher than pre-crisis averages similarly to unemployment rates and expectations.^16^

## 4 Measuring the effect of lock-down measures on online search activities

Daily data on search-based unemployment indicators are complemented with information on policy responses taken by governments to contain the spread of the SARS-CoV-2 virus. In particular, we focus on *shelter-in-place* and otherwise home confinement orders enacted by EU governments as recorded by the Oxford Covid-19 Government Response Tracker (OxCGRT, Hale et al., 2021). We consider as lock-downs country-wide orders requiring not leaving the house with exceptions for daily exercise, grocery shopping and essential trips. According to this definition, we identify 17 countries which enacted lock-down measures: Austria, Belgium, Cyprus, Croatia, Czechia, France, Greece, Hungary, Ireland, Italy, Luxembourg, the Netherlands, Poland, Portugal, Romania, Slovakia and Spain.^17^ Data, including the daily SVI of the best predictors, are collected from the 13th of January to the 30th of June.

**Fig. 4:**
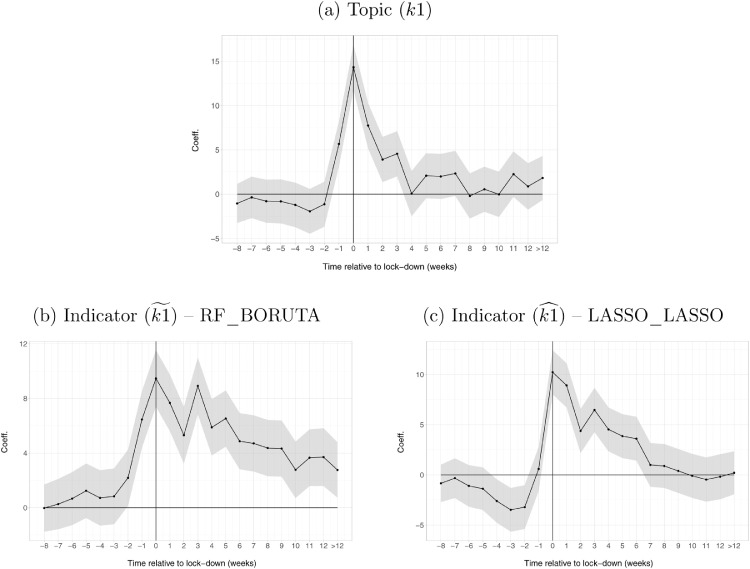
DiD coefficients for k1, $\tilde{k1}$ and $\hat{k1}$. *Note:* panel (a) presents the estimates of Eq. (4) for the topic k1 “unemployment”. Panel (b) and panel (c) present the estimated of Eq. (4) for the indicators $\tilde{k1}$ constructed, respectively, drawing from the set of keywords selected by RF_BORUTA and LASSO_LASSO.

Our DiD regression can be written as follows:

(4) $$y_{c,t}=\alpha +\sum_{\tau =-8}^{12}\beta_{\tau }D_{c,w+\tau }+\beta_{\tau^{+}}D_{c,w+\tau^{+}}+\mu_{c}+\delta_{t}+\varepsilon_{c,t},$$

where the generic term $y_{c,t}$ corresponds to either: $k1_{c,t}$, ${\tilde{k1}}_{c,t}$ or ${\hat{k1}}_{c,t}$. $k1_{c,t}$ is the daily SVI of the topic; ${\hat{k1}}_{c,t}$ is the daily SVI of the indicator obtained through the LASSO_LASSO procedure; and ${\tilde{k1}}_{c,t}$ is the daily SVI of the indicator obtained through the RF_BORUTA procedure, all in country c at time t. $D_{c,w+\tau }$ are 21 relative week dummies centered around the dates of lock-down, meaning that τ=0 in the lock-down week.^18^ $D_{c,w+\tau^{+}}$ is a dummy for weeks greater than 12 which is added to avoid the latter being included in the baseline; $\mu_{c}$ are country fixed-effect and $\delta_{t}$ are date fixed-effect. The inclusion of a set of pre-lock-down dummies is used to provide evidence on the validity of the DiD identifying assumption. Estimates are reported in Fig. 4.

Fig. 4 (a), (b) and (c) shows the results for k1 (a), $\tilde{k1}$ (b) and $\hat{k1}$ (c). All three variables exhibit an increase in the weeks following the announcements of lock-downs. Stringent measures such as the shelter-in-place orders recorded in Eq. (4) sparkled fear of unemployment at the onset of the covid crisis. While the effect on k1 is relatively short-lived, the opposite is true for $\tilde{k1}$ and $\hat{k1}$, where the higher level of unemployment-related queries persists throughout the whole lock-down period. This is particularly true for $\tilde{k1}$.

**Fig. 5:**
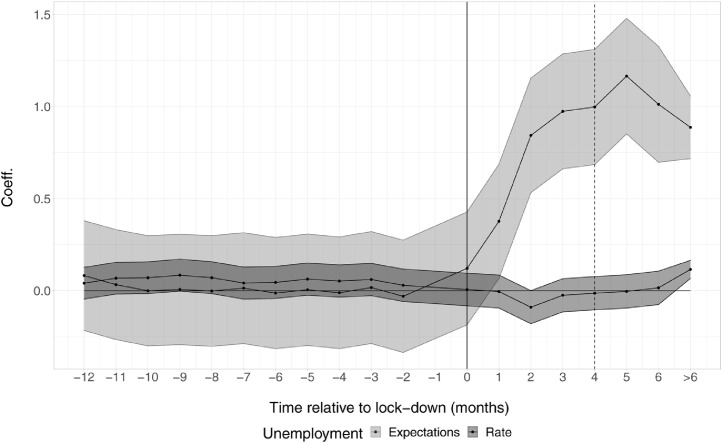
The effect of lock-downs on unemployment rates and expectations. *Note:* The graph shows the coefficients of a DiD regression similar to the one in Eq. (4). The dependent variables considered are: the seasonally adjusted monthly unemployment rate (*ei_lmhr_m*, Eurostat); and the seasonally adjusted monthly unemployment expectations (*ei_bsco_m*, Eurostat). Each series is standardised to have mean 0 and variance 1. Data span from January 2015 to May 2021. The dotted line indicates the end of the time coverage of the daily unemployment indicators (Fig. 4).

To assess which of these measures of unemployment-related web searches (k1 or the ones constructed using the best predictors $\tilde{k1}$ and $\hat{k1}$) better capture labour market dynamics in the wake of the covid-19 pandemic, we conduct a retrospective DiD exercise using monthly official statistics. We estimate two separate equations similar to the one described in Eq. (4) using available monthly data on unemployment rates and the unemployment expectations indicator from January 2015 to May 2021. The set of estimated coefficients are presented in Fig. 5.

The figure shows that, while there is no differential effect on the unemployment rate, also due to the effect of labour market measures enacted by EU Governments, countries that introduced lock-downs exhibit a drastic and time-persistent increase in expectations about the future level of unemployment.

In light of this evidence, the results of the DiD obtained using our constructed indicators suggest that the latter better reflect the differential evolution of expectations rather than that of the unemployment rate. The time-persistence also suggests that, in differences, unemployment expectations dynamics are better mirrored by the constructed indicators than by the topic alone that shows no differential effect after just four weeks.

This holds true despite the fact that, as aforementioned, the expectations indicator does not fully capture the average individuals’ expectations of becoming unemployed in the near future. Rather, it reflects generalized pessimistic views about future labour market conditions in each country. Yet, previous evidence shows that this type of generic and qualitative surveys can convey information about the unemployment rate (Abberger, 2007, Claveria, Pons, Ramos, 2007) and overall economic conditions (Bachmann et al., 2013).

Our findings for the EU27 compare favourably to those by Aaronson et al. (2020) for the US. Aaronson et al. (2020) show that unemployment-related queries surged before the record increase in unemployment insurance claims, which peaked before the lock-down measures were implemented. Our findings suggest that measures introduced by Governments to contain the pandemic generated a negative effect on EU citizens’ economic prospects. Overall, our results also corroborate recent research showing that search data are particularly responsive to sudden labour market shocks (Borup et al., 2020b), offering a timely and almost real-time alternative to official statistics. However, their performance crucially hinges on their association with the underlying phenomenon of interest.

## 5 Conclusion

Researchers are increasingly exploiting online search activities to study phenomena for which timely and high-frequency data are not readily available. In this paper, we propose a data-driven procedure which solves the issue of identifying and combining the list of queries linked to the underlying phenomenon of interest. The resulting indicator can then be used for causal inference.

Exploiting Google Trends topics, we retrieve over four-thousand search queries related to unemployment in the EU27 in their native languages. Then, in the first step of the procedure, using machine learning techniques, we select the search queries that best predict unemployment in each EU country. In the second step, we combine these queries and create search-based unemployment indicators.

Finally, using a DiD approach, we document both the topic and the indicators dynamics in the weeks following the announcements of lock-downs. Overall, stringent measures are linked with increased searches for unemployment-related queries. While the effect on the topic is short-lived, the opposite is true for the indicators constructed using our proposed procedure. Using official statistics, we show that the latter better captures the time-persistence of worsening labour market prospects. This indicates that web search data, if treated correctly, can provide useful insights on labour market dynamics following sudden shocks even in a cross-country perspective.

Importantly, the procedure described in this paper is not only relevant in the context of unemployment nor restricted to the case of the covid-19 pandemic. It could be used to study a variety of events, policies and economic indicators, especially when administrative or survey data are not timely available and/or comparable. In particular, the procedure perfectly fits scenarios in which Google Trends data are used in a multi-language and multi-institutional context. Further, while we use the obtained indicator as a dependent variable, it can be also used on the right-hand side of the estimating equation.

## Declaration of Competing Interest

None

## Figures and Tables

**Fig. A.1:**
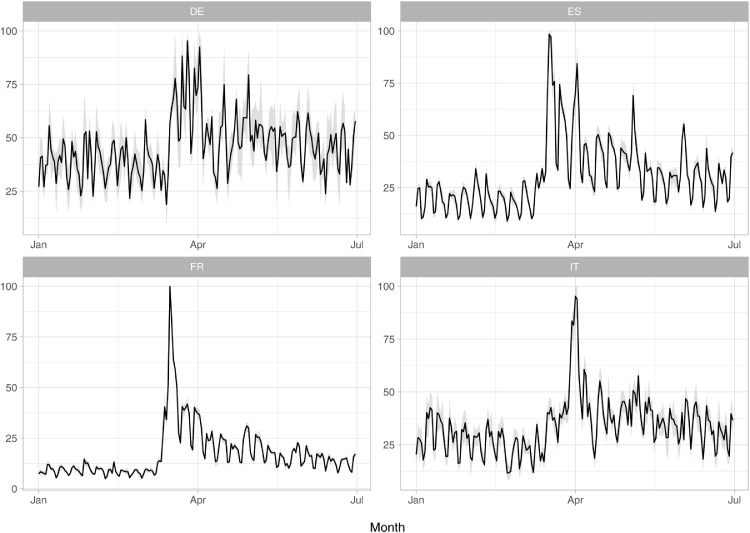
Google searches: sampling variability. *Note:* Each panel contains the average SVI for the topic “unemployment” (/m/07s_c) – black line and the minimum and maximum bandwidth recorded during the five data extraction performed.

**Fig. A.2:**
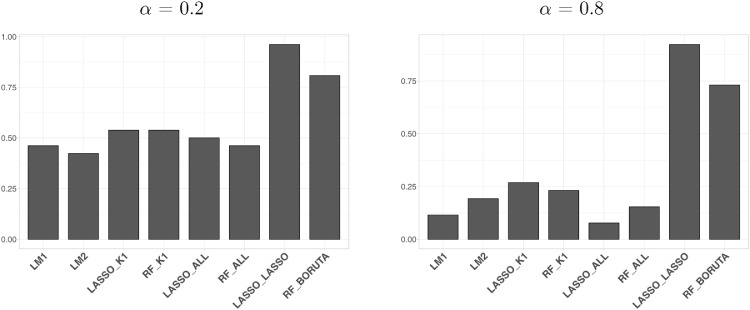
MCS: robustness to different values of α. *Note:* Each bar represents the faction of countries in which the model is retained in the final set according to MCS.

**Table A.1:** List of selected keywords.

Country	Keyword	Country	Keyword
AT	brutto netto rechner	IE	cso
AT	kredit online	IE	cso ireland
AT	ams linz	IE	unemployment
AT	arbeiterkammer	IT	assegni familiari
BE	unemployment	IT	indeed
BG	борса учебници	IT	indennitá di clientela
BG	джобс	IT	inps malattia
BG	зддфл	IT	istat
BG	noi	IT	licenziamento giustificato motivo oggettivo
CZ	zaméstnanost	IT	licenziamento oggettivo
CZ	hdp na obyvatele	IT	accompagnamento
CZ	nezaméstnanost	IT	per chiedere la disoccupazione
CZ	nezaméstnanost v krajích	IT	regione lombardia
DE	arbeitslosengeld berechnen	IT	calcolo disoccupazione
DE	kredit rechner	IT	ccnl
DE	kündigung arbeitgeber	LT	darbo birza
DE	ab wann arbeitslos melden	LT	jonines
DK	kontanthjælp satser	LT	kucios nedarbo diena
DK	navnebetydning	LT	lapkricio 2 nedarbo diena
DK	a kasse	LT	vasario 16
EE	касса по безработице	LT	ar kucios nedarbo diena
EE	cvonline	NL	uwv
ES	cursos online para desempleados	NL	vacatures
ES	cursos sepe	NL	werk
ES	empleo madrid	NL	werken
ES	inem cursos	NL	bijstandsuitkering
ES	junta de andalucia	NL	cbs de bron
ES	paro cita previa	NL	frankrijk
ES	pedir cita previa	PL	przecęitne wynagrodzenie gus
ES	cita previa paro	PL	regon
ES	cursos desempleados	PL	wskaźnik gus
ES	cursos inem	PL	inflacja gus
FI	rauman seudun työttömät	PL	nip
FI	te keskus	PT	cursos profissionais
FI	oaj työttömyyskassa	PT	irs desempregados
FI	päiväraha	RO	indemnizatia de somaj
FI	rakennusliitto	RO	ajofm prahova
FR	convention rupture conventionnelle	RO	baza de calcul somaj
FR	france	SE	eurovision
FR	indemnité congés payés	SE	eurovision 2013
FR	lettre de demission	SE	föräldrapenning
FR	lettre de démission	SE	if metall
FR	rsa	SE	scb
GR	dikaiologitika	SE	seko
GR	κoινωνικoστoυρισμoσ	SE	sommarjobb arbetsförmedlingen
HR	narodne novine	SE	arbetslösheten
HR	posao	SE	bidrag invandrare
HR	croatia	SE	bnp
HR	hzzz	SI	otroški dodatek
HR	isplata dječjeg doplatka	SI	zavod za zaposlovanje
HR	mirovinsko	SI	zzzs maribor
HU	álláskeresési járadék összege	SK	všeobecná zdravotná
HU	munkanélküli hivatal	SK	dôvera
HU	munkanélküli segély mikor jár	SK	otvaracie hodiny urad prace

Notes: Example of selected keywords. Only the keywords selected both by the Lasso and Boruta procedures are shown.

**Table A.2:** Number of selected keywords.

Country	Total	Boruta	Lasso	Country	Total	Boruta	Lasso
AT	159	9	59	IE	101	6	5
BE	199	1	32	IT	378	38	53
BG	189	11	51	LT	82	7	33
CY	16	0	0	LU	23	0	0
CZ	211	6	62	LV	60	0	12
DE	383	11	67	MT	1	0	0
DK	148	9	29	NL	180	26	18
EE	24	2	6	PL	210	19	34
ES	322	30	71	PT	174	3	68
FI	254	7	75	RO	138	4	19
FR	380	17	72	SE	217	14	61
GR	298	5	32	SI	68	3	17
HR	186	12	62	SK	167	4	9
HU	122	4	14				

Notes: Total shows the total number of related keywords retrieved for each country. Boruta and Lasso shows, respectively, the number of keywords selected by the Boruta and Lasso algorithms.

**Table A.3:** Results of the Diebold-Mariano test for equal predictive accuracy comparing different nowcasting models against the benchmark auto ARMA model.

Country	LM2	LASSO_k1	RF_k1	LASSO_ALL	RF_ALL	LASSO_LASSO	RF_BORUTA
AT	−0.008	−0.045**	−0.005	−0.014	−0.02	−0.191***	−0.039
BE	−0.02*	−0.015*	0.001	−0.011	−0.008	−0.046**	0.024
BG	0.007	0.009	0.011	0.001	0.012	−0.045**	−0.009
CY	0.073	−0.009	−0.19**	−0.002	−0.077*	0.006	−0.215***
CZ	−0.001	−0.018	−0.02	0.014	−0.02	−0.027	−0.033*
DE	0.004	0.014	−0.01	0.019	0.014	−0.067***	0.006
DK	−0.027**	−0.06***	−0.039**	−0.046**	−0.02**	−0.091***	−0.055***
EE	−0.017	−0.044**	−0.006	−0.001	−0.018	−0.056	−0.05**
ES	0.029	0.021	−0.054**	−0.002	0.012	−0.079***	−0.033
FI	−0.039	−0.018	0.004	0.048	−0.026	−0.328***	−0.05
FR	0.012	−0.003	−0.026	0.053	−0.038**	−0.113***	−0.095***
GR	0.038	−0.142**	−0.067	−0.017	0.029	−0.262***	−0.171***
HR	−0.004	0.024	−0.028	0.073	0.038	−0.09**	−0.054*
HU	0.008	−0.019*	−0.002	0.042	−0.047**	−0.027	−0.037*
IE	−0.028*	−0.029**	−0.025*	−0.02	−0.012	−0.03*	−0.034***
IT	−0.168**	−0.144**	−0.198***	−0.178**	−0.192***	−0.354***	−0.268***
LT	0.01	−0.016	0.004	−0.032	−0.075***	−0.146***	−0.106***
LU	−0.003	0.001	0.002	0.001	0.005	0.001	0.006
LV	0.006	0.006	0.03	0.011	0.027	−0.008	0.054
NL	−0.016*	−0.027***	−0.035**	−0.078***	−0.082***	−0.105***	−0.097***
PL	0.002	−0.004	−0.014	−0.013	−0.018	−0.046***	−0.031*
PT	0.014	−0.012	−0.009	−0.01	−0.006	−0.086***	−0.048***
RO	0.012	0	0.018	0.01	0.008	0.007	0.01
SE	0.054	0.029	−0.002	0.122	−0.026	−0.198***	−0.055
SI	0.01	0	0.02	0.015	0.022	−0.022	−0.042**
SK	0.001	−0.005	−0.007	−0.015*	−0.023**	−0.028**	−0.036**

*Notes:* Each cell represents, separately for country and each horizon, the difference $g\left(e_{mod.i}\right)-g\left(e_{LM1}\right)$. The loss function used is the absolute deviation, i.e. $g\left(e_{mod.i}\right)=E(|y_{t}-\hat{y_{t,mod.i}}\left|\right)$. *, **, and *** denote significance of the difference at the 10, 5, and 1 percent level, computed according to the one-sided Diebold-Mariano test for predictive accuracy.

**Table A.4:** Results of the Model Confidence Set for equal predictive accuracy comparing different nowcasting models.

Country	LM1	LM2	LASSO_K1	RF_K1	LASSO_ALL	RF_ALL	LASSO_LASSO	RF_BORUTA
AT	✗	✗	✓	✗	✗	✗	✓	✗
BE	✗	✓	✗	✗	✗	✗	✓	✗
BG	✗	✗	✗	✗	✗	✗	✓	✓
CY	✗	✗	✗	✓	✗	✗	✗	✓
CZ	✓	✓	✓	✓	✓	✓	✓	✓
DE	✗	✗	✗	✓	✗	✗	✓	✗
DK	✗	✗	✓	✗	✗	✗	✓	✗
EE	✓	✓	✓	✓	✓	✓	✓	✓
ES	✗	✗	✗	✓	✗	✗	✓	✗
FI	✗	✓	✗	✗	✗	✗	✓	✗
FR	✗	✗	✗	✗	✗	✗	✓	✓
GR	✗	✗	✗	✗	✗	✗	✓	✓
HR	✗	✗	✗	✗	✗	✗	✓	✓
HU	✗	✗	✗	✗	✗	✓	✓	✓
IE	✗	✓	✓	✓	✓	✓	✓	✓
IT	✗	✗	✗	✗	✗	✗	✓	✓
LT	✗	✗	✗	✗	✗	✗	✓	✓
LU	✓	✓	✓	✓	✓	✓	✓	✓
LV	✓	✗	✗	✗	✗	✗	✓	✗
NL	✗	✗	✗	✗	✗	✗	✓	✓
PL	✗	✗	✗	✓	✓	✓	✓	✓
PT	✗	✗	✗	✗	✗	✗	✓	✓
RO	✓	✓	✓	✓	✓	✓	✓	✓
SE	✓	✗	✗	✓	✓	✓	✓	✓
SI	✗	✗	✗	✗	✗	✗	✓	✓
SK	✗	✗	✗	✗	✗	✓	✓	✓

*Notes:* ✓indicates that model m is retained in the final set of selected models for country c. The level of significance for the MCS is α=0.5.

## Footnotes

2 Google excludes from the sampling queries made by very few people; duplicate searches – i.e., queries made by the same individual over a short period; queries containing special characters; and illegal search activities, such as automated searches performed by bots.

3 Topics were introduced by Google in late 2013 for the US and in the following years for EU countries. See https://developers.google.com/knowledge-graph for additional information.

4 We chose the 13th of January as the starting date because (i) it is past the Christmas’ holidays period, which might influence online search behaviour but (ii) it is before the events and the lock-down of Wuhan (23rd January) which might have influenced individuals’ economic expectations.

5 In Fig. A.1 we show that even the topic “unemployment” itself show some sampling variability across the four largest European economies.

6 See Eurostat, Digital Economy and Society Data https://ec.europa.eu/eurostat/web/digital-economy-and-society/data/database.

7 From an empirical perspective, the unemployment rate series well describes the current level of unemployment $U_{t}$ in each EU country. Conversely, the unemployment expectations indicator plotted in Fig. 1 measures the degree of agreement with a question about the overall unemployment situation in each given country over the next 12 months. No harmonised series about individual expectations of becoming unemployed at t+1 ($\delta_{t}$) is instead available. In addition, the current unemployment rate implicitly embeds information about the realisation of future expectations – as also described by the inclusion of ${\tilde{\delta }}_{t-1,t}$ in Eq. (1). Therefore, to retrieve the set of queries describing the latent variable $\omega_{t}^{*}$, we rely on the unemployment rate time series only.

8 The number of related keywords is entirely driven by the Google algorithm linking each web search with a given topic. The difference in the number of related keywords depends on several factors. For example,countries might have different web search behaviour, e.g., a country might have a single or few policies related to unemployment, while another might have more or changed them over time. Besides, the number of retrieved keywords also depends on the search volume and, therefore, to the internet penetration and the size of each country.

9 There are 84 data-points in the original time series (i.e., 12 observations for 7 years). However, differencing and using lags lead to the loss of 4 observations for each time series.

10 See Medeiros et al. (2019) for a comparison of the performance of different machine learning methods in a forecast race targeted at predicting US inflation using a wide set of covariates.

11 For a detailed description of Random Forest see Hastie et al. (2009)

12 Table A.1 provides a sample of the selected keywords. In particular, we show the keywords that have been selected by both Boruta and Lasso algorithms. Table A.2 provides instead (i) the total number of queries retrieved for each country; and (ii) the number of queries selected by either the Boruta or Lasso algorithms.

13 MCS is an iterative procedure in which at each step the hypothesis of equal accuracy of the full set of models is tested. If the hypothesis is rejected at the jth-step, the worse model is excluded from the list and the test is repeated until the hypothesis is not rejected. Results are robust to the different level of α and are presented in Fig. A.2 in the appendix.

14 The choice of absolute deviations instead of the common squared deviations is driven by the scale of our response variable. Using absolute deviations implicitly assign the same weight to each error avoiding to reward those that are particularly small.

15 The keywords that best mimic the unemployment rate have been selected using monthly data. Our indicator uses instead daily data. However, the semantic association between the selected keywords and the underlining phenomenon of interest (the unemployment rate or unemployment expectations) is not likely to depend on the frequency in which they are analysed (e.g., monthly, weekly or daily). Put differently, it is reasonable to expect that if individuals searching for unemployment-related information use query i in month m, they would use the same query in any given day in month m.

16 The figure for $\hat{k1}$ shows an almost identical pattern and is available under request.

17 The dates considered are those of the measure’s announcement: Austria 16-03; Belgium 18-03; Cyprus 24-03; Croatia 23-03; France 17-03; Greece 23-03; Hungary 27-03; Ireland 28-03; Italy 10-03; Luxembourg 17-03; the Netherlands 23-03; Poland 31-03; Portugal 19-03; Romania 25-03; Slovakia 08-04; and Spain 14-03. Malta is excluded from our sample due to the unavailability of related keywords.

18 This does imply that we have an unequal number of dummies before (−8) and after (+12) the lock-downs. The choice of stopping at -8 is because the first country experiencing a lock-down (Italy) only has its first full observable week at -9, which we consider as baseline level for that country.
